# Emerging contaminants: assessing the release of pharmaceuticals via managed aquifer recharge

**DOI:** 10.1007/s11356-025-36536-8

**Published:** 2025-05-22

**Authors:** Dibyanshu Dibyanshu, Traugott Scheytt

**Affiliations:** Department for Hydrogeology and Hydrochemistry, Institute of Geology, Technische Universitat Bergakademie Freiberg, Freiberg, Germany

**Keywords:** Managed aquifer recharge, Pharmaceuticals, Transport, Unsaturated, Saturated

## Abstract

Managed aquifer recharge (MAR) is a vital water management strategy that infiltrates surface water or wastewater effluent into aquifers through soil and sediment. However, this process can introduce pharmaceutically active compounds (PhACs) and their metabolites, posing environmental risks. This study investigates the transport behavior of four selected PhACs—caffeine, carbamazepine, diclofenac, and ibuprofen—under neutral pH conditions using column experiments in both unsaturated and saturated porous media. PhACs and a tracer solution were introduced into the system, and experimental results were simulated using the CXTFIT model to determine retardation and degradation factors. Experimental findings indicate high mobility for carbamazepine and ibuprofen across both unsaturated and saturated conditions with high recovery (> 88%) and negligible degradation (< 0.05). Ibuprofen behaved similarly identical to the tracer in unsaturated medium and with small adsorption/desorption in saturation with a retardation factor of ~ 1, while carbamazepine showed slight retardation and tailing effects showing higher persistence in the water, with high dispersion in unsaturated medium. Diclofenac significantly degrades in saturated media (45% recovery) but increases release under unsaturated conditions (91% recovery). This indicates the release of diclofenac through the vadose zone but can undergo degradation and retardation in aquifers. Caffeine exhibited high retardation and degradation, with a low recovery rate (27–30%) under both conditions, suggesting that degradation or irreversible adsorption may have occurred independently of moisture content during transport. This finding highlights the differences in mobility and degradation across unsaturated and saturated zones, posing long-term contamination risks of PhACs. This underscores the need for robust monitoring and management to prevent groundwater contamination from pharmaceuticals, pathogens, and antibiotic-resistant bacteria, especially when using wastewater effluent or stormwater for recharge.

**Graphical Abstract:**

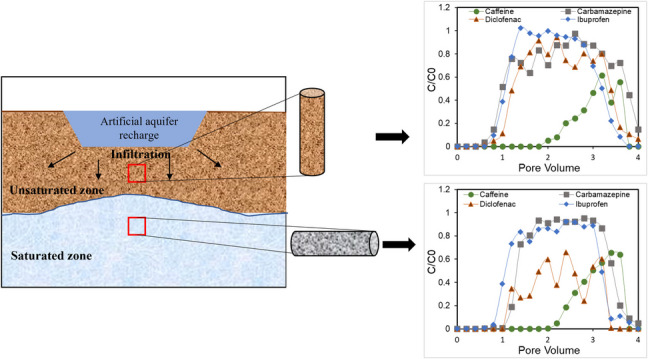

## Introduction

The increase in population, climate change, and economic growth has increased the concern about water scarcity in many countries worldwide, especially in places like arid and semi/arid regions. Managed aquifer recharge (MAR) is one of the integral parts of water and wastewater management strategies that aim to restore aquifers using excess water resources like rainwater or treated wastewater (Maeng et al. 2011; Nham et al. 2015; Silver et al. 2018). It is accomplished by infiltration through the continuum of unsaturated zone-saturated zone, which appears as one of the major solutions to the recurrent issue of water scarcity. The wastewater effluent, which might be used for the MAR includes emerging contaminants like pharmaceutical active compounds (PhACs). PhACs are reported in wastewater and surface water at a wide range, ranging from ng/L to mg/L (Balakrishna et al. 2017; Dibyanshu et al. 2024; Guruge et al. 2019; Keerthanan et al. 2021; Maeng et al. 2011; Sim et al. 2010; Waleng and Nomngongo 2022). When such effluents are used in MAR to enhance groundwater supplies, there is a risk of these contaminants infiltrating through the unsaturated–saturated zone and causing a risk of groundwater contamination. The unsaturated zone, serving as a natural filter, may partially attenuate some contaminants, but many still reach the saturated zone, posing risks to groundwater quality (Pedersen et al. 2005). Few studies have investigated the occurrence of PhACs in the groundwater receiving infiltrated treated wastewater (Nham et al. 2015; Patterson et al. 2011; Williams and McLain 2012). Even though the effect on human health has not yet been proven for the detected concentration range (ng/L to μg/L), the increasing detection of wastewater-originated compounds is seen to be problematic for a safe water supply, also under ethical aspects. Understanding the transport, fate, and potential removal mechanisms of these emerging contaminants during MAR is crucial. This knowledge helps in designing effective recharge systems that minimize environmental and health impacts, ensuring that groundwater remains a safe and sustainable resource for future use.

Laboratory-scale experiments are frequently conducted under controlled environment conditions to determine the fate and potential exposure of PhACs during infiltration. Experimental data can be simulated using numerical and/or analytical modelling of coupled transport to obtain reactive transport parameters. Most of the column experiments available in the recent literature have been performed under saturated conditions to simulate the transport of these compounds during aquifer recharge, agriculture irrigation, and soil aquifer treatment (Heberer and Adam 2004; Hebig et al. 2017; Kiecak et al. 2019; Labad et al. 2023; Mehrtens et al. 2022; Schübl et al. 2021; Scheytt et al. 2004; Williams and McLain 2012). Saturated conditions simulate scenarios where water fills soil pores, promoting specific geochemical and microbial processes affecting PhAC transport. These processes are influenced by factors like oxygen availability and soil properties, which are key for degradation and sorption behaviors. However, under unsaturated conditions, where water only partially fills soil pores, the fate of PhACs differs significantly. Unsaturated zones, characterized by variable oxygen availability, affect microbial activity and chemical degradation pathways. Few studies have been conducted on the transport behavior of PhACs under unsaturated conditions (Koroša et al. 2020; Martínez-Hernández et al. 2017; Scheytt et al. 2006, 2007). However, there is a lack of information on its transport behavior via the infiltration-based MAR method, where water moves through the unsaturated zone before entering the saturated aquifer. This process influences the transport, retention, and degradation of contaminants, making it crucial for safe and sustainable aquifer recharge. Existing literature, including Bouwer (2002) and Gorski et al. (2020), highlights the significance of MAR and the need to understand unsaturated flow dynamics under both unsaturated and saturated conditions.

**Fig. 1:**
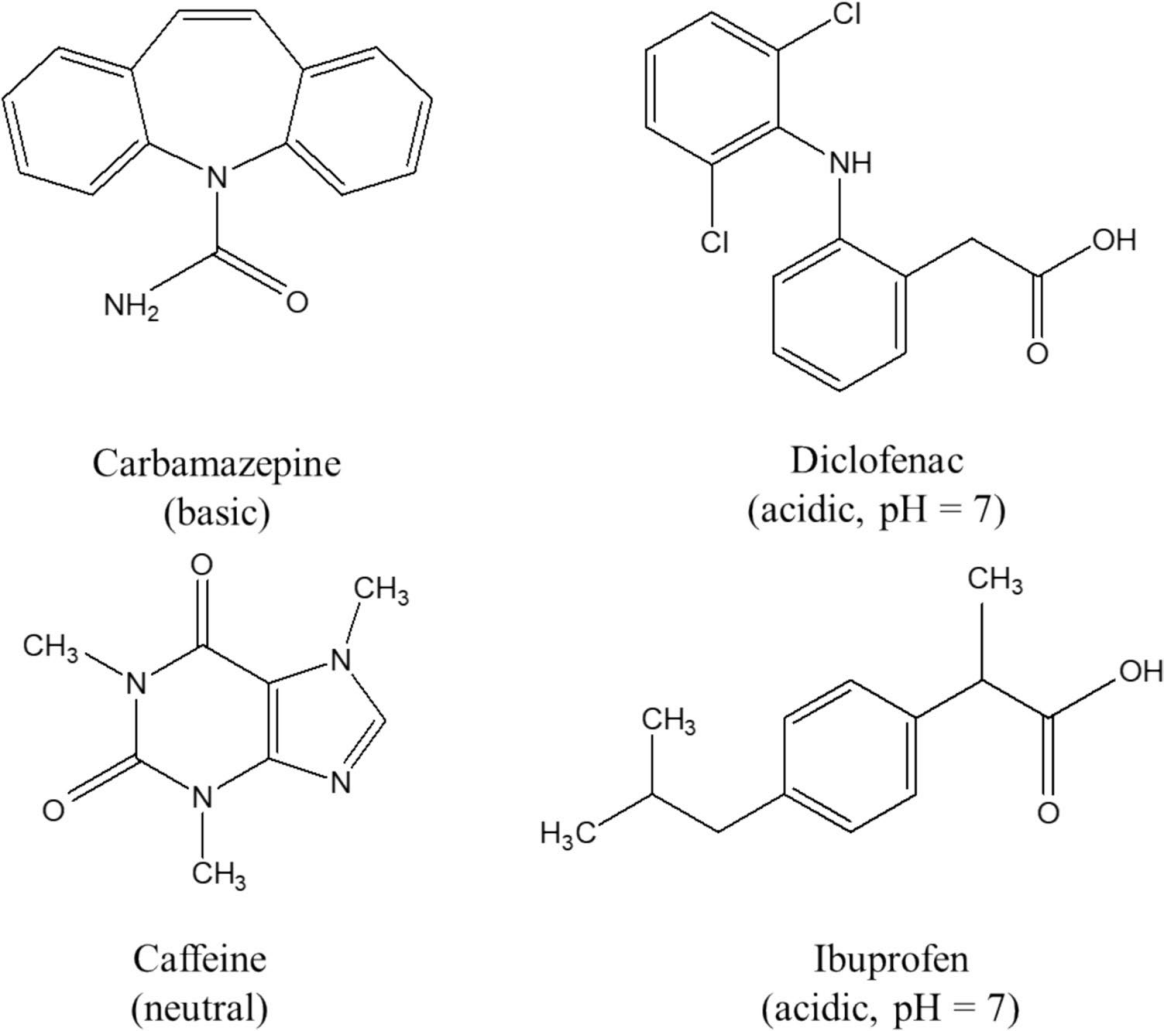
Molecular structure of the selected trace compounds

Therefore, the objective of this study is to examine the infiltration of PhACs in MAR systems, coupled with reactive modeling to predict the fate of contaminants. Specifically, it examines the behavior of four commonly detected PhACs—caffeine, carbamazepine, diclofenac, and ibuprofen—as they migrate through the vadose zone under unsaturated conditions and into the aquifer under saturated conditions. The study employs specially designed laboratory equipment to replicate key transport processes, providing insights into their mobility, retention, and degradation. Caffeine, widely used in beverages, foods, and pharmaceuticals, is selected for its prevalence, while carbamazepine, an antiepileptic drug, is known for its persistence in water. Diclofenac and ibuprofen, both anti-inflammatory drugs, are chosen due to their toxicity and global occurrence. The experiment was conducted at a neutral pH, where carbamazepine and caffeine are in neutral form while diclofenac and ibuprofen are negatively ionized (Fig 1, Table 1). In addition to evaluating the transport dynamics, the study also aims to understand the environmental risks associated with the release of these PhACs by investigating their pollution sources, the age of contamination, and their potential link to secondary pollution, particularly concerning microbial risks.

**Table 1:** Physical and chemical properties of trace compounds

Compounds	Use	Solubility (mg/L)	Log K_ow_	pKa (20 °C)	Internal standard
Caffeine	Stimulant	2.16 × 10^4^	− 0.07	14	Caffeine-^13^C_3_
Carbamazepine	Anti-convulsant	17.7	2.25	13.9	Carbamazepine-D_10_
Diclofenac	Anti-inflammatory	4.5	4.02	4.16	Diclofenac-D_4_
Ibuprofen	Anti-inflammatory	21	4.02	4.52	Ibuprofen-D_3_

Values listed here are from Koroša et al. (2020) and Silver et al. (2018)

## Methodology

### Materials and sample preparation

The PhACs used in this study, such as caffeine, carbamazepine, diclofenac, and ibuprofen, are obtained from Sigma-Aldrich. The study used reagent-grade potassium nitrate (KNO_3_) and sodium chloride (NaCl) as a tracer solution. Methanol, acetonitrile, and formic acid used for the measurement in liquid chromatography-mass spectrometer (LC–MS/MS) instruments are of HPLC grade. The organic-free sediment used for the column study was purchased from the OBI market in Freiberg.

The 1 g/L stock solution of different PhACs was prepared by mixing 0.02 g of individual PhAC powder in 20 ml of methanol. From the stock solution, the concentration of 100 µg/L was prepared by adding 100 µl of stock solution to 1000 ml of deionized (DI) water. The pH of the solution is maintained at 7 ± 0.2 using 1 N NaOH and 1 N HCl solution.

### Column transport experiment

#### Characterization of porous media

The sediment for the column experiment was purchased from the OBI market and had a grain size between 0.4 and 0.7 mm. The sieve analysis was done to check the grain size composition. The sand was then washed with DI water to remove impurities and afterwards dried in the oven at 100 °C for 24 h. With this, we expect the sediment to be essentially organic-free. To evaluate the porosity of the sediment, first, the column was wet-packed with the sediment used as porous media. Once the column was completely saturated, the saturated sediment was extracted in a 4-cm section from the bottom end of the column by injecting DI water from the top end of the column at a very low flow rate. The wet weight of the extracted sediment from the column was measured and then dried for 24 h at a temperature of 100 °C to measure the dry weight of the sediment. Then the effective porosity (${\varepsilon }_{eff}$) was calculated, which was found to be around 0.42.

#### Laboratory setup

A plexiglass column (3-cm inner diameter, 30-cm length) was used to conduct both unsaturated and saturated column transport experiments to evaluate the behavior of selected PhACs. Organic-free sand was used as the porous medium and was pre-saturated with DI water for 24 h to ensure uniform conditions.

**Fig. 2:**
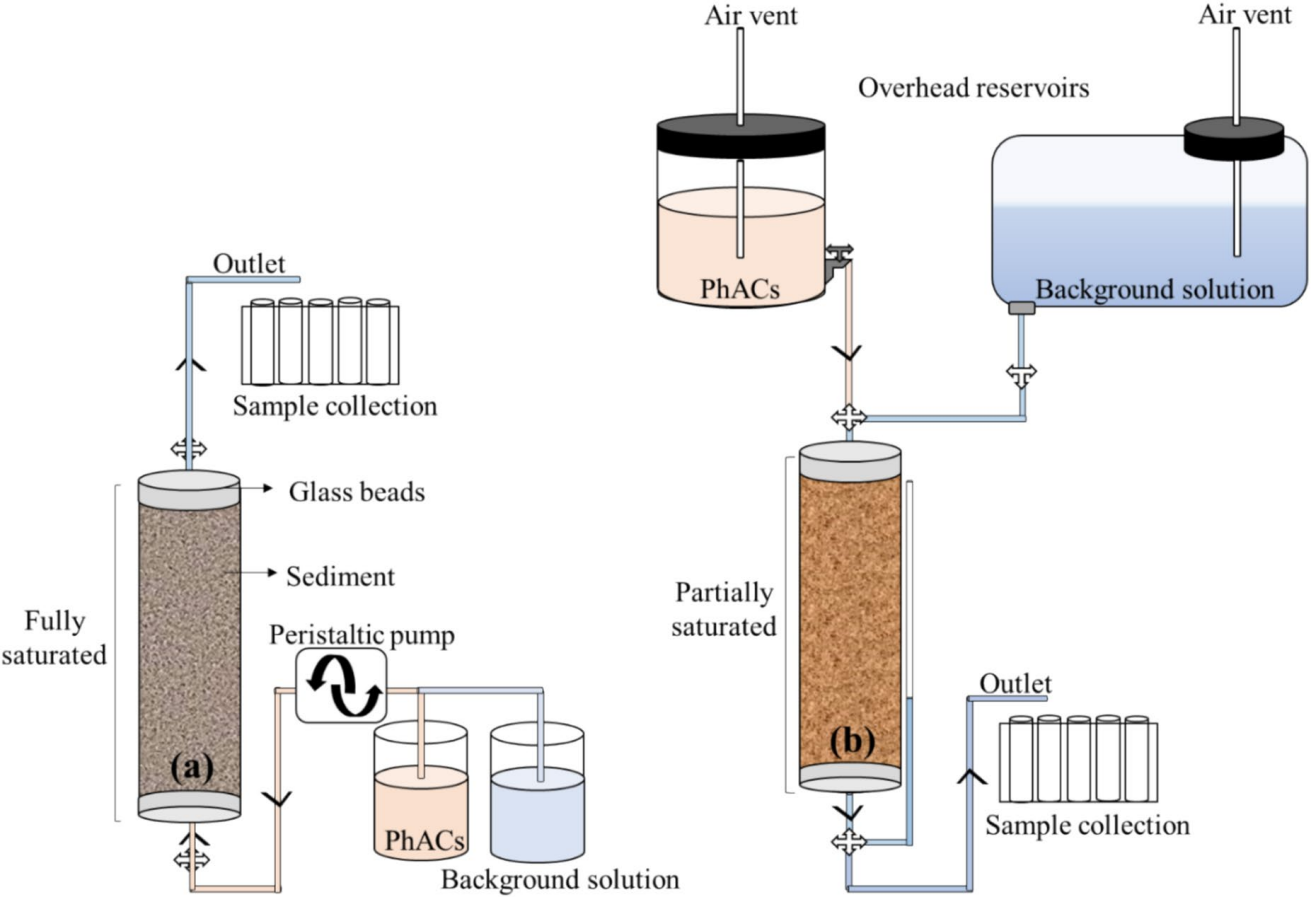
Schematic diagram illustrating the column setup for transport experiments: **a** saturated conditions with upward flow, and **b** unsaturated conditions with downward flow

The sand was packed using the wet packing method, where incrementally saturated sand was added and compacted uniformly with a tamping rod. A 10-mg/L KNO₃ solution was used as a conservative tracer to analyze water movement in the column. The solution was introduced upward from the column’s bottom using a peristaltic pump to eliminate air bubble entrapment, maintaining a flow rate of 1.4 mL/min. The selected flow rate of 1.4 mL/min aligns with reported MAR infiltration rates when scaled to laboratory conditions (Bouwer 2002; Gorski et al. 2020). This ensures a balance between realistic field conditions and controlled experimental precision for reliable assessment of PhAC transport and degradation. After injecting the tracer for two pore volumes (PVs), the column was flushed with DI water for an additional 2 PV. Figure 2a shows the schematic diagram of the experimental setup of the saturated column experiment. Effluent samples were collected regularly to evaluate breakthrough curves and water movement behavior. The PhAC transport experiments were conducted in a similar manner, injecting 100 µg/L solutions of caffeine, carbamazepine, diclofenac, and ibuprofen, followed by DI water flushing. Effluent samples were collected at regular intervals, ensuring consistent flow and tracking compound movement through the column.

For the unsaturated setup, the column transport occurred downward under gravity using a Mariotte bottle to maintain a constant hydraulic head (Fig. 2b). The column was packed using the same wet packing method as in the saturated experiments. To achieve unsaturated conditions, the column bottom valve and top air vent valve were opened, allowing air to enter and water to drain naturally under gravity, leaving the lower ~ 4 cm of the column saturated. A separate tube at the column bottom maintained the water level, ensuring steady conditions as shown in Fig. 2b. Once drainage was stabilized, the air vent was closed, and the background solution valve was open. The constant flow of water (approximately 1.4 mL/min) was achieved by adjusting the height of the effluent tube, which approximately matches the water-level tube. This method created variable moisture content conditions that mimic the vadose zone. To determine water flow behavior during unsaturated conditions, the PhAC solutions were spiked with a 1 mM NaCl solution (serving as a tracer). The movement of chloride ions helped validate water transport patterns through the unsaturated media. The PhAC solution with tracer was injected for 2 PV, followed by 2 PV flushing with the background solution (DI water). Regular effluent samples were collected at controlled time intervals to analyze breakthrough curves and observe PhAC transport behavior under unsaturated conditions.

All experiments were conducted under controlled conditions to compare PhAC transport behavior at neutral pH in unsaturated and saturated systems, including flow consistency checks throughout the experiments. The two setups—upward flow for saturated conditions and downward flow under gravity for unsaturated conditions—ensured an accurate evaluation of tracer and PhAC mobility through the porous media.

#### Sample analysis

Samples collected from the column transport experiment were divided into two portions. One portion was filtered with a 0.45-µm membrane filter and was stored in centrifuge tubes. This was used for both cation and anion analysis using an ion chromatography (IC) instrument. The second portion was placed in glass vials for measuring the concentrations of selected PhAC (i.e., caffeine, carbamazepine, diclofenac, and ibuprofen) using an LC–MS/MS (Sciex EXION LC and Sciex QTRAP 6500 + MS) instrument. The analysis of the PhACs was performed in the Hydrogeochemistry laboratory of Ruhr University Bochum.

The analysis of PhAC concentrations was performed following the procedure described by Dibyanshu et al. (2024). In brief, PhACs were analyzed using an LC–MS/MS instrument, employing internal standards for calibration and analysis. These standards included Carbamazepine D_10_, Caffeine ^13^C_3_, Diclofenac D_4_, and Ibuprofen D_3_, all obtained from Sigma-Aldrich with a purity of ≥ 99%. For the analysis, 1 mL of the collected samples was mixed with 20 μL of the internal standard using a vortex in HPLC vials. It was then centrifuged at 10,000 rpm for 10 min at 8 °C using an IKA mini-G. These HPLC vials were placed in an autosampler, and the method for analyzing the selected PhACs was run. The method includes chromatographic separation using a C18 column (Phenomenex Synergi 4 μm Fusion-RP, 80 Å LC Column, 50 × 2 mm) at a flow rate of 0.40 mL/min, with an oven temperature of 50 °C and an injection volume of 50 μL. The eluents used were water with 2 mM NH_4_F and 0.01% formic acid (eluent A) and methanol (eluent B), both of LC–MS grade. The gradient method started with 10% eluent B for 0.5 min, increased to 95% over 7 min, held at 95% for 8.5 min, and returned to 10% for the final 8.7 min. Electrospray ionization (ESI) was used with a spray voltage of 5.5 kV in positive mode and − 4.5 kV in negative mode, a temperature of 500 °C, GS1 and GS2 set at 70 and 50, respectively, and a curtain gas of 35 V. The caffeine, carbamazepine, and diclofenac were measured in positive mode while ibuprofen was measured in negative mode. The limit of quantification for the selected compounds is 0.66 µg/L (caffeine), 0.0002 µg/L (carbamazepine), 0.0052 µg/L (diclofenac), and 0.188 µg/L (ibuprofen).

### Modelling the transport of PhACs in sand column experiments

The movement, retention, and transformation of pollutants in porous media are governed by a complex interplay of physical and chemical processes. Understanding these mechanisms is critical for predicting the fate of pharmaceuticals in subterranean environments. Numerical modelling of tracer breakthrough curves and estimation of transport parameters was performed using the STANMOD software CXTFIT 2.0 (Tang et al. 2010; Scheytt et al. 2007). Deterministic equilibrium and inverse mode based on the convection–dispersion equation (CDE) (Tang et al. 2010) were tested to fit the transport models to the measured data. These models take into account the retardation caused by sorption processes, which have an impact on the contaminant’s movement and distribution. The CDE one-dimensional steady-state flow in the homogeneous subsoil is presented in Eq. 1, according to Tang et al. (2010), where C [ML^−3^] is the normalized concentration (*C*/*C*_0_) in the fluid phase at distance *x* [L] and time *t* [T], *R*_f_ is the retardation coefficient, *D* denotes the hydrodynamic dispersion coefficient [L^2^T^−1^], υ stands for the average pore water velocity [υ = q/ε_eff_, where *q* is the Darcy velocity and ε_eff_ is the porosity, [LT^−1^] and μ [T^−1^] is the first-order degradation coefficient resulting from degradation.

The advection–dispersion equation (ADE) with a retardation factor *R*_f_ is expressed as:

1 $${R}_{f}\frac{\partial C}{\partial t}=D\frac{{\partial }^{2}C}{\partial {x}^{2}}-\upsilon \frac{\partial C}{\partial x}-\mu C$$

The initial step involves estimating the water transport parameters (pore velocity υ and the dispersion coefficient, *D*) using nitrate as a conservative tracer for saturated porous media and chloride for unsaturated media (Tables 1 and 2). The effluent concentration data obtained from the column experiment are fitted as an inverse problem using the deterministic equilibrium ADE mode, considering flux-averaged concentrations and dimensional time and positions. For this, the retardation factor (*R*_f_) is set to 1.0, and the first-order degradation coefficient (μ) is fixed at 0. In the model, the initial velocity (ν) is obtained from the column flow rates, while dispersion coefficients (*D*) are calculated as longitudinal dispersion coefficients (*D*_*L*_) using the equation:

2 $${D}_{L}={\alpha }_{L}\upsilon$$

where *D*_L_ is the longitudinal dispersion coefficient [L^2^ T^−1^]; α_L_ is the longitudinal dispersivity [L]. Studies have shown that dispersivity is the intrinsic property of the porous media under fully saturated conditions, while the greater value of dispersivity is observed for the same media under unsaturated conditions (Bromly and Hinz 2004; Jin et al. 2000). Factors contributing to these variations include the presence of immobile water regions, a broader range of pore water velocities in unsaturated media, and increased tortuosity of solute pathways due to air-filled pore spaces (Bromly and Hinz 2004).

**Table 2:** Parameters for unsaturated column transport experiment

Column	Q (ml/min)	ɵ	ɛ_eff_	*S*_d_
Caffeine	1.2	0.149	0.42	0.355
Carbamazepine	1.2	0.150	0.42	0.357
Diclofenac	1.6	0.143	0.42	0.340
Ibuprofen	1.4	0.153	0.42	0.364

ɵ: volumetric water content; ɛeff: effective porosity; *S*_d_: degree of saturation

The parameters *D* and ν derived from the conservative tracer breakthrough curves are then used as constants to fit the reactive breakthrough of PhACs. These fitted velocities and dispersions are used to determine the retardation factor (*R*_*f*_) and degradation coefficient (µ) for the selected PhACs. However, the analytical solution to the ADE, as presented by Bromly and Hinz (2004), was also used to obtain retardation factors (*R*_*f*_):

3 $${R}_{f}=\frac{{\upsilon }_{water}}{{\upsilon }_{contaminant}}=1+\frac{{\rho }_{b}}{\theta }{K}_{d}$$

where ρb is the bulk density [M L^−3^], *ɵ* is the volumetric water content, and *K*_d_ is the sorption distribution coefficient defined as the ratio of the concentration of the substance in the solid phase (*C*_sorb_) to the concentration in water (*C*_*w*_) at equilibrium:

4 $${K}_{d}=\frac{{C}_{sorb}}{{C}_{w}}$$

This distribution coefficient was derived from column experiments using the above equation, referred to as the transport distribution coefficient *K*_*d*_. For all pharmaceutical breakthrough curves, the maximum *C*/*C*_0_ value is used to indicate the amount eliminated via biological and chemical degradation. Mass balance for the pharmaceuticals is calculated by subtracting input mass from output mass, with output mass obtained through graphical integration of the breakthrough curve areas and is mentioned in percentage recovery of PhACs through the column.

## Results and discussions

### Transport behavior of conservative tracer

**Fig. 3:**
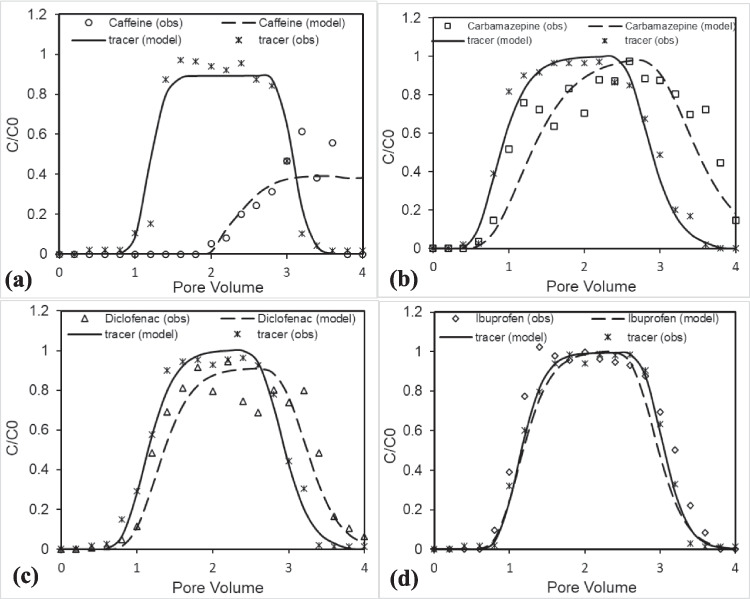
Transport behavior of **a** caffeine, **b** carbamazepine, **c** diclofenac, and **d** ibuprofen in unsaturated porous media at pH 7. Symbols represent experimental data, and lines show model simulations

**Fig. 4:**
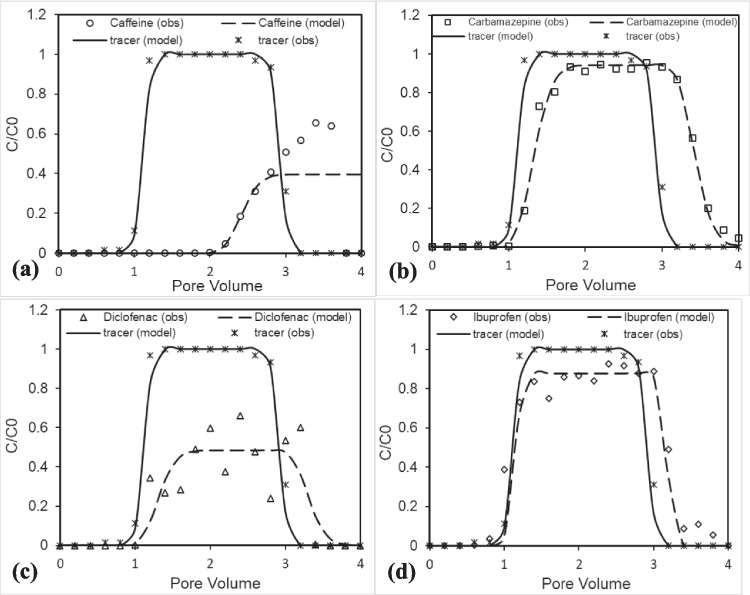
Transport of **a** caffeine, **b** carbamazepine, **c** diclofenac, and **d** ibuprofen in saturated porous media at pH 7. Symbols represent experimental data, and lines show model simulations

A conservative tracer study was conducted to evaluate water movement through saturated and unsaturated porous media. In saturated media, nitrate served as the tracer, injected independently, while in unsaturated media, chloride was spiked with the PhAC solution during injection. The tracer fitting for both conditions was done using the CXTFIT model, represented in Figs. 3 and 4. An excellent fit was obtained with an *R*^2^ value of more than 0.95, which confirms that the transport is mainly affected by chemical equilibrium but not due to diffusion. The model fitting of chloride in the unsaturated media exhibits significantly higher dispersion coefficients, ranging from 0.116 cm^2^/min (caffeine) to 0.890 cm^2^/min (carbamazepine), resulting in broader breakthrough curves (Fig. 3, Table 3). The heterogeneous flow paths due to variations in water saturation and movement lead to variability in the dispersion coefficient. In contrast, saturated conditions showed a lower dispersion of 0.044 cm^2^/min, reflecting a steep breakthrough curve (Fig. 4), due to uniformly filled pore spaces devoid of air pockets, facilitating more homogeneous flow. A study by Bromly and Hinz (2004) also demonstrated higher dispersivity under unsaturated conditions compared to saturated conditions. The absence of early tracer release in saturated conditions suggests a negligible preferential flow path and promotes more uniform transport through the media, while unsaturated conditions create preferential flow pathways where some regions experience faster movement while remaining stagnant. Consequently, the observed differences in dispersion among caffeine, carbamazepine, diclofenac, and ibuprofen are likely influenced by the degree of water saturation and the resulting heterogeneous flow dynamics rather than solely by the inherent properties of the pharmaceuticals. Recovery rates of the conservative tracers exceeded 92%, with recoveries of 96% in saturated conditions and 92–99% in unsaturated conditions (e.g., 92% for carbamazepine and 99% for diclofenac, Table 3). Velocity estimates from model fitting were approximately 0.503 cm/min for saturated conditions and 0.465–0.485 cm/min for unsaturated conditions, demonstrating slightly slower flow under unsaturated conditions. These findings highlight distinct water movement dynamics influenced by moisture content and help validate the experimental approach for understanding solute transport in porous media.

**Table 3:** Conservative transport parameters determined by fitting the BTC of nitrate tracer for the saturated column and chloride tracer for the unsaturated column

	Saturated column	Unsaturated column			
	Selected PhACs	Caffeine	Carbamazepine	Diclofenac	Ibuprofen
*R*_f_ (fixed)	1.0	1.0	1.0	1.0	1.0
µ [day^−1^] (fixed)	0.0	0.0	0.0	0.0	0.0
*D*_*fitted*_ [cm^2^ min^−1^]	0.044	0.116	0.890	0.389	0.399
*υ*_*fitted*_ (cm/min)	0.503	0.465	0.485	0.480	0.480
*R*^2^	0.99	0.952	0.973	0.986	0.989
Mass recovery (%)	96	93	91	99	96

*R*_f_: retardation factor; µ: degradation coefficient; *D*_fitted_: dispersion; *υ*_fitted_: flow velocity

### Transport behavior of selected pharmaceuticals

**Fig. 5:**
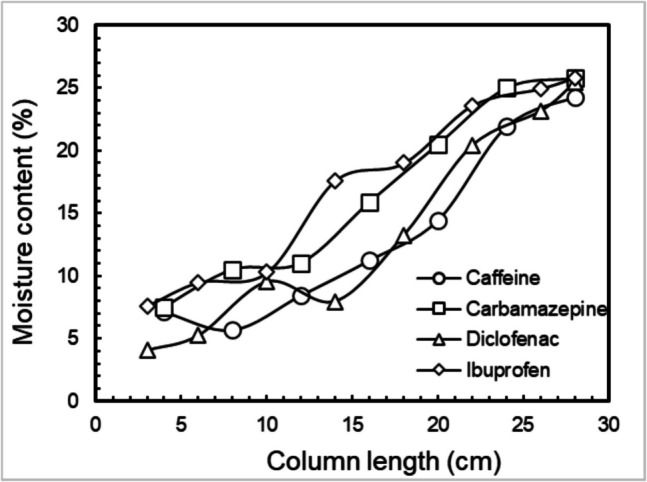
Moisture content throughout the column during transport through unsaturated porous media

The transport behavior of four target PhACs—caffeine, carbamazepine, diclofenac, and ibuprofen—through unsaturated and saturated porous media was investigated at pH 7 using chloride and nitrate as tracers for water movement. The moisture content within the unsaturated porous media was also checked by dismantling the column after the freezing-melting process and measuring its wet and dry weight. Figure 5 shows that for all the unsaturated experiments, the moisture content in the column ranged from 5 to 25%, which increased with an increase in the distance from the injection point, reflecting vadose zone conditions. The degree of saturation, ranging from 0.34 to 0.36, also indicated uniform saturation in all the column experiments (Table 2). The findings reveal varying degrees of interaction and transport for each compound, influenced by their physicochemical properties and environmental interactions. The transport experiment was conducted at a flow rate of 1.4 ml/min in saturated media using a peristaltic pump, while in unsaturated media, the flow rate was 1.4 ml/min with a fluctuation of ± 0.2 ml/min under constant head conditions. The flow rates recorded for each unsaturated column setup are presented in Table 2. The effluent samples from all the experiments were collected at 10-min intervals (approx. 0.2 PV) and were measured using LC–MS/MS.

#### Caffeine

The transport behavior of caffeine studied in both unsaturated and saturated porous media at neutral pH shows significant similarities in their breakthrough curve, as shown in Figs. 3a and 4a. In both scenarios, caffeine exhibited a delayed breakthrough curve releasing at 2 PV compared to the conservative tracer, with *R*_f_ of ~ 2.18–2.19 and µ of ~ 0.360 day^−1^ (unsaturated) and ~ 0.384 day^−1^ (saturated) (Table 4) obtained from the model fit. The high values of *R*_f_ and µ indicated that caffeine is highly sorbed and degraded during its transport. Recovery was comparable, around ~ 29.8% in the unsaturated system and ~ 30.4% in the saturated system, indicating limited differences in caffeine behavior across moisture regimes. However, its recovery in the observed data shows a slight increase from 26.7% in unsaturated media to 30.5% in saturated media. Caffeine’s transport exhibited delayed release, driven by its interactions with the negatively charged porous matrix due to its basic nature, having a neutral or positively charged state at pH 7 (pKa ~ 14). However, its subsequent release during water flushing was primarily influenced by its high hydrophilicity (log Kow ~  − 0.07), which reduces its affinity for the porous medium and facilitates desorption. Its desorption following water flushing resulted in a peak concentration of *C*/*C*_0_ ~ 0.504 for unsaturated and 0.656 for saturated conditions (Table 4), followed by slower declines that might be attributed to microbial degradation.

**Table 4:** Summary of parameters of column experiment

Column	Observed Data		Model Data						
	*C/C*_0_	Recovery (%)	C/C_0_	Recovery (%)	*v *(cm/min)	D(cm^2^/min)	*R*_f_	µ (day^−1^)	*R*^2^
**Unsaturated column**									
**Caffeine**	0.504	26.7	0.392	29.8	0.465	0.116	2.18	0.360	0.71
**Carbamazepine**	0.896	97.1	0.975	89.3	0.485	0.890	1.19	0.000	0.82
**Diclofenac**	0.802	91.3	0.909	98.0	0.480	0.389	1.17	0.048	0.91
**Ibuprofen**	0.948	101.1	0.998	90.0	0.480	0.399	1.03	0.000	0.91
**Saturated column**									
**Caffeine**	0.656	30.5	0.396	30.4	0.503	0.044	2.19	0.384	0.59
**Carbamazepine**	0.953	92.0	0.942	90.6	0.503	0.044	1.20	0.024	0.99
**Diclofenac**	0.660	44.8	0.483	44.3	0.503	0.044	1.20	0.312	0.77
**Ibuprofen**	0.924	88.5	0.912	85.3	0.503	0.044	1.02	0.048	0.96

The high retardation and notable degradation observed in both conditions are consistent with previous studies highlighting caffeine’s substantial attenuation in porous media through adsorption and biotransformation (Martínez-Hernández et al. 2017; Koroša et al. 2020). Studies on a field scale were also conducted, showing high removal of caffeine during infiltration (Conn et al. 2010). However, the occurrence of caffeine in groundwater (Dibyanshu et al. 2024) is likely due to its high consumption and its use in beverages, food, and pharmaceuticals. The results show caffeine sensitivity to environmental interactions while underscoring its consistent behavior in different moisture conditions.

#### Carbamazepine

The breakthrough curve for carbamazepine in the unsaturated media (Fig. 3b) demonstrates a slightly delayed arrival compared to the tracer, indicating that carbamazepine experiences retardation as it moves through the porous media. This retardation is due to interactions between carbamazepine and the porous media, likely driven by adsorption mechanisms. Its moderate hydrophobicity (Log Kow ~ 2.25) and neutral charge at environmental pH (pKa ~ 13.9) contribute to its tendency to adsorb onto the porous media. Additionally, the breakthrough curve of carbamazepine is more spread out and exhibits a noticeable tailing effect compared to the sharp rise and fall observed for the tracer. This tailing indicates a broader range of residence times, reflecting both adsorption–desorption equilibrium processes and non-linear sorption. These processes slow the transport of carbamazepine through the porous media and contribute to its retention. The broader curve of carbamazepine, which indicates a slow release of the compound during transport, has also been observed in other literature (Hebig et al. 2017; Martínez-Hernández et al. 2017). Despite these interactions, it shows a high recovery rate of approximately 97.1% from observed data (Table 4), with negligible degradation observed during the experiment. This high recovery is likely due to the reversible sorption of the compound, as the absence of organic matter in the organic-free sand minimizes strong sorption interactions, allowing adsorption to be dominated by weak electrostatic or hydrogen bonding mechanisms. The comparable value of *R*_f_ (~ 1.19–1.20), negligible µ, and recovery (89.3–90.6%) in both the unsaturated and saturated conditions indicate that the water content does not significantly impact the transport of carbamazepine (Table 4). However, the tailing effect in the saturated condition (Fig. 4b) is comparatively less than observed in the unsaturated condition (Fig. 3b), likely due to the presence of air-filled pores, which influence the adsorption and desorption of carbamazepine during transport. These observations underscore carbamazepine persistence and potential for long-distance transport in subsurface environments. The high persistence of carbamazepine was also reported by Williams and McLain (2012) when measuring its concentration in groundwater monitoring wells receiving water from the wastewater recharge basin with about 2 years of travel time.

#### Diclofenac

The breakthrough curve of diclofenac in unsaturated porous media shown in Fig. 3c demonstrates transport behavior similar to carbamazepine, characterized by delayed breakthroughs relative to the conservative tracer, indicating retardation (*R*_f_) value around 1.17. This is attributed to adsorption–desorption interactions with the porous matrix, showing a recovery of around 91.3% (observed data, Table 4). This behavior can be attributed to its acidic nature (pKa ~ 4.2) and moderate hydrophobicity (log Kow ~ 4.51). At neutral pH, diclofenac exists primarily in its anionic form, reducing its adsorption to the media compared to non-ionic compounds. However, its hydrophobic interactions contribute to a moderate degree of retardation. The desorption-driven tailing is indicative of a non-linear sorption process, which slows down contaminant flushing.

In saturated conditions, diclofenac exhibits a gradual increase in concentration starting around 1 PV, peaking at a relative concentration (*C*/*C*_0_) of 0.660 in the observed data and 0.483 in the modeled curve (Fig. 4c, Table 4). This behavior reflects significant interactions with the porous medium, likely driven by diclofenac’s hydrophobic nature and low water solubility (2.37 mg/L), which enhance its retention on the media’s surface. The slow desorption observed after 2 PV underscores these retention dynamics, leading to a recovery rate of only 44.8%, substantially lower than the 91.3% noted under unsaturated conditions (Table 4). Additionally, the degradation rate increases from 0.048 day⁻^1^ in unsaturated conditions to 0.312 day⁻^1^ in saturated conditions (Table 4). This higher degradation rate in saturated media can be attributed to enhanced hydrolysis and increased microbial activity facilitated by the high moisture content. Saturated conditions provide an ideal environment for both chemical and biological degradation processes to occur more effectively. The differences in diclofenac transport between unsaturated and saturated conditions arise from increased degradation and stronger adsorption in saturated media, coupled with limited desorption due to higher water content. These findings highlight the significant role of media saturation in diclofenac transport, emphasizing greater retention and slower movement in saturated environments compared to unsaturated conditions.

#### Ibuprofen

The behavior of ibuprofen in unsaturated porous media (Fig. 3d) demonstrates a transport pattern nearly identical to that of a conservative tracer, as evidenced by the close alignment between observed and modelled data. This suggests minimal retardation of ibuprofen in the porous media, allowing it to move with minimal interaction with the media itself. The weak interaction between ibuprofen and the porous matrix is crucial to this observation. Despite having a higher hydrophobicity (with a Log Kow of 4.02) compared to other compounds such as carbamazepine and caffeine (Table 1), the ionization characteristics of ibuprofen significantly influence its behavior. The pKa value of ibuprofen is approximately 4.52, meaning that at environmental pH, it exists predominantly in its anionic form. This anionic form of ibuprofen results in reduced adsorption to the porous media, further facilitating its mobility through the medium. As a result, it exhibits minimal retardation (*R*_f_ ~ 1.03) and negligible degradation during transport, leading to a recovery rate of 101% (observed data, Table 4). In contrast to these findings, Scheytt et al. (2004) observed significant retardation and reduced recovery of ibuprofen when it was introduced as artificial sewage effluent and transported through an unsaturated sand column. However, when transported through saturated media, ibuprofen experiences only a slight reduction in recovery (85.3%) compared to its recovery in unsaturated media (90.0%) in model-fitted data (Fig. 4d, Table 4), while at observed data the difference is slightly more with 88.5% for saturated conditions and 101.1% for unsaturated conditions (Table 4). This difference in recovery rates is attributed to its significantly higher water solubility (21 mg/L) in contrast to less soluble compounds, which minimizes retention within the porous medium and enhances its mobility, leading to a higher recovery rate in unsaturated conditions. These results underscore ibuprofen as a highly mobile contaminant in unsaturated porous media, where it moves almost like a conservative tracer.

## Conclusion and environmental implications

MAR is a vital water management strategy for utilizing treated wastewater or stormwater to replenish groundwater resources, particularly in water-scarce regions. However, it also presents risks of transporting pollutants deep into the subsurface, potentially contaminating groundwater. This study investigated the fate and transport of four pharmaceutical compounds—caffeine, carbamazepine, diclofenac, and ibuprofen—during MAR through infiltration. A column experiment was conducted under both unsaturated and saturated conditions at neutral pH to assess their mobility.

The findings of this study reveal significant insights into the transport and environmental implications of pharmaceuticals detected in MAR systems using stormwater and wastewater effluent. To assess degradation pathways, the sediment used in the experiments was oven-dried at 100 °C for 24 h, likely eliminating most indigenous microorganisms. However, water and other experimental components were not sterilized. Therefore, degradation was assumed to be primarily driven by microorganisms introduced through the infiltrating water. The observed mobility, persistence, and interactions of the pharmaceutical compounds with the porous media provide critical information on potential pollution sources, contamination timelines, and associated microbial risks. Carbamazepine and ibuprofen demonstrated high mobility under both, unsaturated and saturated conditions, with minimal interaction with the porous media. Carbamazepine’s behavior is attributed to its high solubility, moderate hydrophobicity, and also its high persistence in water. Its transport exhibited a tailing effect—marked by a broader breakthrough curve—reflecting slow and extended-release influenced by adsorption–desorption dynamics. Such behavior has been similarly observed for other organic micropollutants like parabens, whose fate in aquifer materials is strongly influenced by changes in water chemistry and sorption equilibria (López-Ortiz et al. 2018). This indicates that carbamazepine can release slowly through the subsurface once introduced. Its persistence underscores long-term contamination risks from wastewater effluent, pointing to ongoing pollution sources. Moreover, the presence of carbamazepine suggests potential microbial risks, as such persistent pharmaceuticals can foster the development of antibiotic-resistant bacteria by exerting selective pressure on microbial communities (Andrade et al. 2020; Buelow et al. 2021). Additionally, it can help trace the pathways of microorganisms introduced into the environment. This increase in antibiotic-resistant bacteria contaminates drinking water sources, posing significant health risks to humans and wildlife. Furthermore, it perpetuates a cycle of resistance, undermining global efforts to manage infectious diseases effectively. Ibuprofen, characterized by its high solubility and anionic form at neutral pH, showed behavior similar to that of a conservative tracer. Electrostatic repulsion between ibuprofen and the negatively charged porous media enhances its mobility, allowing it to infiltrate groundwater systems with limited retardation. The limited degradation of ibuprofen is also observed due to the absence of microbial interaction. However, other studies show rapid degradation of ibuprofen both, in aerobic and anaerobic conditions (Langenhoff et al. 2013), where the microbial activity is robust and also due to its relatively short environmental half-life (Koumaki et al. 2017). The detection of ibuprofen in aquifers, despite its rapid degradation in aerobic and anaerobic environments, points to recent pollution events such as sewer system leaks, untreated wastewater infiltration or stormwater runoff. If there were no recent inputs, ibuprofen would likely degrade and become undetectable over time. These incidents may also introduce microbial pathogens like *Escherichia coli*, which can survive and proliferate in aquifers, posing public health risks. The ibuprofen, once it reaches the aquifer, will be highly toxic with time as some of its degradation products are more toxic than the original product (Ellepola et al. 2020), and also if the amount of degradation products is very high (Hussain et al. 2024). In contrast, diclofenac exhibited varied mobility depending on environmental conditions. While highly mobile under unsaturated conditions, its movement is significantly reduced in saturated environments due to its lower water solubility and greater interaction with porous media. This suggests that diclofenac can infiltrate through the vadose zone but accumulates near recharge areas within the groundwater system. Diclofenac’s presence indicates contamination from wastewater effluent and agricultural activities such as biosolid application or runoff. This association with agricultural practices raises concerns about microbial contaminants, including zoonotic pathogens and antibiotic-resistant bacteria, entering MAR systems via stormwater (Buelow et al. 2021). Caffeine, a widely recognized marker of urban pollution, demonstrated delayed transport under both unsaturated and saturated conditions. Despite its high solubility and low hydrophobicity, caffeine’s movement is influenced by sorption and degradation, leading to accumulation in porous media. However, contrasting findings from other studies (Hebig et al. 2017; Martínez-Hernández et al. 2017) suggest negligible release of caffeine during transport, indicating its rapid degradation and minimal retardation effect on mobility. This accumulated caffeine on the soil surface is likely to be released into the subsurface during the movement of stormwater. Its persistence in anaerobic environments suggests contamination from municipal sewage systems, with potential ongoing release into groundwater. The presence of caffeine also indicates significant microbial risks, as urban wastewater can harbor a variety of pathogens. The persistence of both caffeine and these pathogens under anaerobic aquifer conditions underscores the dual threat of chemical and microbial contamination in MAR systems that utilize untreated or partially treated wastewater.

Overall, the study underscores the environmental risks of pharmaceuticals in MAR systems. Highly mobile contaminants like carbamazepine and ibuprofen can infiltrate deeply into aquifers, posing long-term risks to groundwater quality. Diclofenac, with its contrasting mobility behaviors, may accumulate near recharge sites, while caffeine’s transport highlights the potential for localized contamination. The presence of these PhACs in the MAR not only reflects varying mobility and degradation behavior through the unsaturated and saturated zone but also signals broader contamination risks in the long term, including the introduction of antibiotic-resistant bacteria and microbial pathogens. These findings reveal the critical need for robust management and monitoring of MAR systems to prevent groundwater contamination from pharmaceuticals, microbial pathogens, and antibiotic-resistant bacteria, particularly when utilizing wastewater effluent or stormwater for aquifer recharge.

## Data Availability

Datasets generated during the current study are available from the corresponding author on reasonable request.
